# MUC16 mucin (CA125) attenuates TRAIL-induced apoptosis by decreasing TRAIL receptor R2 expression and increasing c-FLIP expression

**DOI:** 10.1186/1471-2407-14-234

**Published:** 2014-04-01

**Authors:** Isabelle Matte, Denis Lane, Marianne Boivin, Claudine Rancourt, Alain Piché

**Affiliations:** 1 Département de Microbiologie et Infectiologie, Faculté de Médecine et des sciences de la santé, Université de Sherbrooke, 3001, 12ième Avenue Nord, Sherbrooke, Québec J1H 5N4, Canada

**Keywords:** MUC16/CA125, Mucins, Death receptors, TRAIL, Ovarian cancer, Apoptosis

## Abstract

**Background:**

MUC16 (CA125) is a large transmembrane mucin protein (> 200 kDa) aberrantly expressed in approximately 80% of human epithelial ovarian cancers (EOC). MUC16 expression in EOC cells is associated with increased tumorigenesis and inhibiton of genotoxic drug-induced apoptosis. However, the mechanism by which MUC16 mediates these effects is unknown. In the present study, we investigated the mechanisms by which MUC16 attenuates TRAIL-induced apoptosis.

**Methods:**

MUC16 expression was down-regulated by stably expressing an anti-MUC16 single-chain antibody (scFv) targeted to the endoplasmic reticulum (ER), which prevents cell surface localization of MUC16 in OVCAR3 cells. We also generated a MUC16 C-terminal domain (MUC16CTD) construct that was stably expressed in MUC16 negative SKOV3 cells.

**Results:**

We show that MUC16 attenuates apoptosis, activation of caspase-8 and mitochondria activation in EOC cells in response to TRAIL. MUC16 decreases TRAIL receptor R2 (DR5) expression and inhibits pro-caspase-8 activation at the death-inducing signaling complex (DISC). MUC16CTD expression is sufficient to attenuate the TRAIL signaling cascade. MUC16 knockdown decreases caspase-8 inhibitor cFLIP mRNA levels, increases cFLIP degradation, and consequently increases TRAIL-induced apoptosis. Down-regulation of cFLIP following treatment of MUC16-expressing OVCAR3 cells with cFLIP siRNA also increases TRAIL-induced apoptosis.

**Conclusions:**

These findings indicate that MUC16 protects EOC cells against TRAIL-induced apoptosis through multiple mechanisms including the blockade of TRAIL R2 expression and the regulation of cFLIP expression at both the transcriptional and the protein level.

## Background

Apoptosis plays a critical role in cellular homeostasis and prevents the development of tumor cells. The apoptotic response of cells can be induced by the intrinsic and the extrinsic pathway, the former being mediated by the mitochondria and the latter activated by ligand stimulation of death receptors at the cell surface [1]. Death receptor ligands such as tumor necrosis factor-related apoptosis-inducing ligand (TRAIL) trigger rapid apoptosis *in vitro* and *in vivo* in various tumor cell types [2-7]. TRAIL binds to death receptors, TRAIL-R1 (DR4) and -R2 (DR5), whose cytoplasmic death domain (DD) signals downstream caspase activation to mediate TRAIL-induced apoptosis [8]. In contrast, TRAIL-R3, TRAIL-R4 and osteoprotegerin (OPG) act as decoy receptors [9-11]. Upon receptor activation, FADD and pro-caspase-8 are recruited to form a death-inducing signaling complex (DISC) [12]. When recruited to the DISC, pro-caspase-8 becomes activated and subsequently activates downstream effectors caspases-3, -6 and -7, leading to apoptosis. Pro-caspase-8 activation can directly result in cleavage of caspase-3 to execute apoptosis (type I cells) or cleave Bid to produce a truncated form (tBid), which induces the release of cytochrome c from the mitochondria leading to caspase-9 and subsequent caspase-3 activation (type II cells) as it is the case for EOC cells. The cellular FLICE inhibitory protein (cFLIP) regulates both recruitment and processing of pro-caspase-8 within the DISC [13]. There are two major splice variants expressed in human cells, cFLIP_S_ (25 kDa) and cFLIP_L_ (55 kDa) [14]. Both isoforms are able to block, although via different mechanisms, caspase-8 activation within the DISC. Consequently, cFLIP isoforms are potent negative regulators of the TRAIL signaling cascade.

MUC16 mucin (CA125) is a large transmembrane glycoprotein that shares many characteristics of the membrane-bound mucin proteins [15-18]. Whereas MUC16 expression is found in the majority of EOC of serous type, it is not detected in normal ovarian epithelium [19]. The structure of MUC16 consists of an enormous N-terminal domain with more than 22,000 heavily glycosylated amino acid residues, a central domain containing up to 60 glycosylated repeat sequences constituting the characteristic tandem repeats of mucins and a C-terminal domain (CTD) [15-18]. The MUC16CTD anchors the protein at the cell surface and consists of a 229 amino acid extracellular region containing a potential proteolytic cleavage site, a 23 residue transmembrane domain, and a 31 amino acid cytoplasmic tail. MUC16 extracellular domain binds to mesothelin [20-22], galectin-3 [23] and Siglec-9 [24]. MUC16 may be involved in suppressing natural killer cell activity [25]. Expression of MUC16CTD in malignant cells enhances migration, invasion, tumor growth and metastasis whereas MUC16 knockdown completely abolishes tumor formation *in vitro* and *in vivo*[26]. MUC16 knockdown sensitizes ovarian cancer cells to apoptosis induced by genotoxic drugs [27]. The mechanisms by which the CTD of MUC16 mediates these biological effects are unknown.

In the present study, we show that MUC16 decreases TRAIL-R2 expression, increases cFLIP expression, blocks recruitment of caspase-8 to the DISC, and consequently attenuates the activation of the TRAIL-induced apoptotic pathway.

## Methods

### Cell lines

The OVCAR3 and SKOV3 human ovarian cancer cell lines were obtained from the American Type Culture Collection (Manassas, VA). OVCAR3 cells were grown in RPMI 1640 (Wisent, St-Bruno, QC, Canada) supplemented with 20% heat-inactivated FBS (Wisent), 2 mM L-glutamine (Wisent), 100 units/ml penicillin, 100 μg/ml streptomycin and 10 μg/ml insulin, and maintained at 37°C in a humidified 5% CO_2_ incubator. The SKOV3 cell line was maintained in DMEM/F12 (Wisent) supplemented with 10% heat-inactivated FBS, glutamine andantibiotics. OVCAR3 R350 cells are a TRAIL-resistant isogenic cell line that was obtained by exposing OVCAR3 cells to stepwise increases of TRAIL over 4 months. This cell line has been described previously [28]. The construction of the anti-MUC16 scFv has been previously described in detail [26,27]. Two independent stable OVCAR3 clones expressing the anti-MUC16 scFv (1:9#7 scFv, 1:9#9 scFv), and the control scFv (ctrl scFv) were generated by transfection of these plasmids into OVCAR3 cells and their validation has been described previously [26,27]. Derivation of MUC16CTD-expressing SKOV3 cells has also been previously described [26,27]. In this construct, MUC16CTD is tagged at the C-terminal with an His and a c-myc tag to allow easy detection of MUC16CTD expression.

#### Reagents

Recombinant human TRAIL was purchased from PeproTech, inc. (Rocky Hill, NJ). The TRAIL-R2 agonist antibody (clone 71903), the tetrapeptide caspase inhibitor, z-IETD-fmk, anti-XIAP and anti-caspase-8 antibodies were obtained from R&D Systems (Minneapolis, MN). Anti-caspase-9, anti-caspase-3, anti-Bid, and anti-Bcl-X_L_, anti-mouse HRP and anti-rabbit HRP antibodies were purchased from Cell Signaling (Beverly, MA). TRAIL-Flag and anti-TRAIL-R1 to R4 receptor antibodies used for flow cytometry were from Alexis Biochemicals (San Diego, CA). Anti-FADD and anti-TRAIL R2 used to perform Western blotting were from EMD Millipore (Etobicoke, ON, Canada). Anti-c-FLIP_L_ and anti-c-FLIP_S_ antibodies were purchased from Calbiochem (LaJolla, CA). Anti-Bcl-2 and anti-CA125 M11 antibodies were obtained from Dako (Burlington, ON, Canada) and anti-CA125 OC125 antibody was from Zymed (South San Francisco, CA). XTT, phenazine methosulfate, propidium iodide, cycloheximide, anti-Flag M2 and anti-tubulin were from Sigma (Oakville, ON, Canada). Anti-Bax and anti-myc-789 used for immunoprecipitation experiments were from Santa Cruz Biotechnology Inc. (Santa Cruz, CA). Anti-myc 9E10 antibody used for Western blot detection, real-time PCR Taqman Gene Expression Assay Master Mix, Flip primers for RT-PCR assays and Flip siRNA were from Life Technologies Inc (Burlington, ON, Canada). Anti-His antibody used for immunoprecipitation experiments was from Bioshop Canada (Burlington, ON). RNAse was obtained from Roche (Laval, QC, Canada).

#### Cytotoxicity assays

Cell viability was determined by the XTT assay. Briefly, cells were plated at 20,000 cells/well in 96-well plates. The next day, cells (confluence 60-70%) were treated with human recombinant TRAIL or anti-TRAIL-R2 agonist antibody as indicated and incubated for 48 h. In some experiments, synthetic caspase inhibitor (25 μM z-IETD-fmk) was added 1 h before the addition of 25 ng/ml of TRAIL. At the termination of the experiment, the culture media was removed and a mixture of PBS and fresh media (without phenol red) containing phenazine methosulfate and XTT was added for 30 min. The absorbance of each well was determined using a microplate reader at 450 nm (TecanSunrise, Research Triangle Pack, NC). The percentage of cell survival was defined as the relative absorbance of treated versus untreated cells. All assays were performed in triplicate and repeated three times.

#### Apoptosis assays

Caspase-3 fluorogenic protease assay was performed according the manufacturer’s protocol (R&D Systems, Minneapolis, MN). In brief, 3 × 10^6^ cells were lysed in 250 μl of cell lysis buffer, and total cell lysates were incubated with 50 μM of DEVD-AFC substrate for 1 h. Caspase-3 activity was measured on a Versa Fluor fluoremeter (BioRad, Hercules, CA). Protein concentration of the lysates was measured with Bio-Rad protein assay kit according to the manufacturer’s recommendations.

To determine the sub-G0 DNA content, floating and adherent cells were harvested, washed with PBS/2% FBS and fixed with cold ethanol for 2 h. Cell pellets were resuspended, washed with PBS, filtered on nylon mesh membrane (40 μm mesh) to remove cell aggregates. Cells were then incubated with propidium iodide (final concentration 20 μg/ml in PBS, RNase A (0.5 mg/ml) and 0.1% Triton X100 overnight at 4°C. Cells were analysed on a FACSCAN flow cytometer (Becton Dickinson, Mississauga, ON).

The release of nucleosomal DNA into the cytoplasm as a measure of apoptosis was determined using the Cell Death Detection ELISA kit according to the manufacturer’s instructions (Roche, Laval, QC, Canada). Briefly, cells were lysed and the extracted cytoplasmic nucleosomal DNA was captured in ELISA wells containing anti-histone antibodies. The nucleosomal DNA was detected with an anti-DNA-POD conjugated antibody. The absorbance of each well was determined using a microplate reader at 410 nm (TecanSunrise, Research Triangle Pack, NC). Each sample was assayed in duplicate. Data are from three independent experiments.

The mitochondrial membrane integrity in OVCAR3 controls and knockdown cells was assayed using the MitoLight™ Apoptosis Detection Kit (EMD Millipore). Cells were cultured in Labtek II chamber slides (Nalge Nunc International, Naperville, IL) in RPMI1640 supplemented with 20% FBS at 37°C to achieve 70-80% confluence. Fresh media containing 200 ng/ml (OVCAR3 cells) or 500 ng/ml (SKOV3 cells) TRAIL was then added and cells were incubated at 37°C in 5% CO_2_ for 45-60 min. After treatment with TRAIL, unfixed cells were incubated with the MitoLight™ reagent (Chemicon, Billerica, CA) (dilution 1:1,500) for 20 min at 37°C. The wells were washed and identical fluorescein and rhodamine fields were digitized at 400× magnification using an Olympus X170 fluorescence microscope. Dye uptake by non-apoptotic mitochondria concentrates in the mitochondrial membrane and is visualized as the accumulation of red fluorescence in the organelles. However, apoptotic mitochondria are incapable of accumulating the dye due to loss of membrane potential and therefore the monomeric dye in the cytoplasm appears green.

#### Immunoprecipitation and immunoblot analysis

Whole cell extracts were prepared in lysing buffer containing protease inhibitors and were separated by 12% SDS-PAGE gels. Proteins were transferred to PVDF membranes (Roche, Laval, Québec, Canada) by electroblotting, and immunoblot analysis was performed as previously described (18). All primary antibodies were incubated overnight at 4°C in 5% milk. Proteins were visualized by enhanced chemiluminescence (GE Healthcare, Baie d’Urfé, Québec, Canada). SKOV3 empty vector and SKOV3 MUC16CTD cell lines were lysed for an hour on ice and immunoprecipitates were obtained using anti-HIS, anti-myc-789 or isotypic anti-mouse IgG monoclonal antibodies conjugated to protein-G agarose (EMD Millipore, Billerica, CA). Immune complexes were separated by SDS-PAGE, immunoblotted and probed with anti-c-myc 9E10 antibodies and visualized by enhanced chemiluminescence. For TRAIL DISC analysis, 80% confluent cells were stimulated with 1 μg/ml (OVCAR3) or 2 μg/ml (SKOV3) of Flag-tagged TRAIL and 3 μg/ml of anti-Flag M2 (pre-incubated for 15 min) in RPMI medium for 30 or 60 min. Cells were then washed with ice-cold PBS and lysed with lysis buffer containing 30 mM Tris-Hcl, 150 mM NaCl, 1% Triton supplemented with protease inhibitors. Lysates were cleared, normalized for protein concentration and the DISC were immunoprecipitated with protein G agarose beads overnight at 4°C on a rotating rod. For Western blot analysis, the beads were washed 4 times with lysis buffer and heated in lysis buffer 4×SDS before the supernatants were separated by 12% SDS-PAGE. NIH Image J software was used to quantify the intensity of each band on Western blots. In some experiments, cycloheximide (200 μM) in DMSO was added for the indicated time.

#### RT-PCR analysis

Total RNAs were extracted from Ctrl scFv- and MUC16 scFv-expressing OVCAR3 cells using Trizol (Life Technologies) according to the manufacturer’s recommendations. RNA integrity was verified on gel by ethidium bromide staining and quantification was performed by determining absorbance at 260 nm. For each sample, total RNA (2 μg) and 1 μM of oligo dT (Promega) were incubated for 5 min at 70°C followed by the addition of 90 units of the reverse transcriptase MMULV (Promega, Madison, WI) and 2.5 μM dNTP for 1 h at 42°C. Amplification of cDNA was done in a PCR reaction using the Taqman Gene Expression Assay Master mix from Applied Biosystems as follow: 2 min at 50°C, 10 min at 95°C and then 40 cycles of 15 s at 94°C and 1 min at 60°C on a StepOne Plus real time PCR system (Life Technologies Inc). The primers for the amplification of cFLIP_L_ (Gene expression assay Hs00153439_m1) and cFLIP_S_ (Gene expression assay Hs00354474_m1) were from Life Technologies Inc.

#### Flow cytometry for TRAIL receptors and MUC16 expression

Cell monolayers were detached using EDTA, washed with PBS and fixed with paraformadehyde 4% in PBS for 20 min. Cells were incubated with the following unlabeled primary antibodies (10 μg/ml) for 1 h at room temperature with human anti-TRAIL-R1, -R2, -R3, and -R4 antibodies. The isotypic control antibody was a normal mouse IgG (BD Biosciences, Mississauga, ON). After three washes with PBS, cells were incubated with PE-conjugated donkey anti-mouse antibody (Jackson ImmunoResearch, West Grove, PA) for 45 min at room temperature. Cells were washed three times and analyzed immediately using a FACSCAN flow cytometer (Beckton Dickinson). After they had reached 80-90% confluence, MUC16 knockdown cells and ctrl scFv cells as well as the parental OVCAR3 cells were fixed with 4% paraformaldehyde. Cell surface MUC16 tumor antigen was detected by flow cytometry using the anti-CA125 M11 mouse monoclonal antibody and revealed using an anti-mouse PE-conjugated (Jackson ImmunoResarch).

### Immunofluorescence

Stable OVCAR3 clones expressing the various scFvs were grown on glass slides until a 50-70% confluence was reached. Glass slides were then washed in cold PBS and cells fixed in ice-cold methanol for 10 min at -20°C. Glass slides were next rinsed 5 min in cold PBS, permeabilized in PBS containing 0.1% Triton X-100 for 5 min on ice and rinsed again in PBS. Slides were blocked in PBS/2% BSA on ice for 45 min and incubated with primary antibodies in blocking buffer at room temperature for 1 h. Slides were washed 3 times in cold PBS, incubated for 30 min at room temperature with anti-mouse secondary antibodies coupled to Texas Red (1:1000; Molecular Probes, Eugene, OR), washed with PBS and mounted for visualization by fluorescence microscopy. Expression of MUC16 was detected using M11 antibody (1:500). Nuclear staining performed with DAPI (100 ng/ml) (Roche), was viewed and photographed using an Olympus X170 fluorescence microscope.

#### Statistical analysis

Statistical comparisons between two groups were performed using the Student’s *t*-test and with ANOVA when comparing the data with more than two treatments groups. Statistical significance was indicated by *P* < 0.05.

## Results

### MUC16 attenuates TRAIL-induced apoptosis and pro-caspase-8 activation

**Figure 1:**
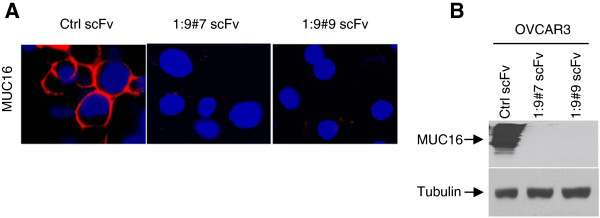
**Cell surface knockdown of MUC16 in OVCAR3 sublines expressing ER-targeted anti-MUC16 scFvs.** Ctrl scFv-expressing cell line and MUC16 knockdown (1:9#7 scFv and 1:9#9 scFv) cell were assessed for MUC16 expression by immufluorescence with anti-CA125 M11 antibody. **(A)** The expression of MUC16 in non-permeabilized cells was determined by immunofluorescence. Cells were incubated with the anti-CA125 M11 antibody and nuclei were stained with DAPI. **(B)** Immunoblot analysis of MUC16 expression. Equal loading was confirmed using an anti-tubulin antibody.

**Figure 2:**
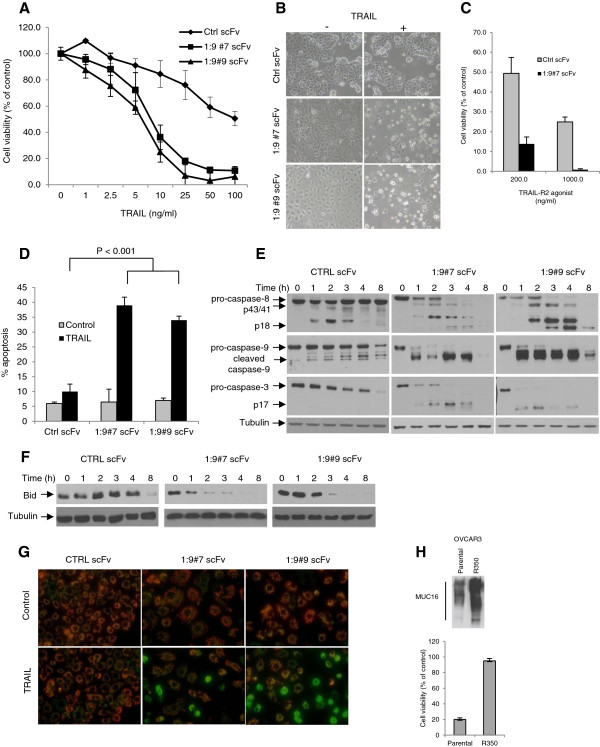
**MUC16 knockdown enhances TRAIL-induced activation of caspase-8 and apoptosis. (A)** Cells were incubated for 48 h with increasing concentrations of TRAIL. Cell viability was measured by the XTT assay. All assays were performed in triplicate and repeated three times. Data are expressed as the mean of pooled data from all three experiments: *bars*, ± SEM. **(B)** Cells were incubated in the presence or absence of TRAIL (100 ng/ml) for 24 h, and morphological changes were assessed by phase contrast microscopy. Representative fields from one of two independent experiments are shown. **(C)** Cells were treated with anti-TRAIL-R2 agonist for 48 h. Cell viability was measured by the XTT assay. All assays were performed in triplicate and repeated three times. Data are expressed as the mean of pooled data from all three experiments: *bars*, ± SEM. **(D)** Cells were treated with TRAIL (100 ng/ml) for 24 h and stained with PI, and the subG0/G1 DNA content was analyzed by flow cytometry. Results from two independent experiments are shown. **(E)** Cells were treated with TRAIL (100 ng/ml) for up to 8 h and cell lysates were analyzed by immunoblots. **(F)** Cells were incubated with TRAIL for the indicated times and cell lysates were immunoblotted with anti-Bid antibody. The anti-Bid antibody used does not detect the truncated form of Bid. **(G)** Cells were cultured for 24 h without TRAIL and the mitochondrial membrane integrity was assessed using MitoLight™. In treated cells, fresh culture medium containing 200 ng/ml of TRAIL was added for 45 min prior to MitoLight™ staining. Cells containing red-labeled mitochondria are scored as viable cells. **(H)** OVCAR3 and isogenic TRAIL resistant R350 cells were treated with TRAIL (100 ng/ml) for 48 h and cell viability was assessed by XTT. Immunoblot showing expression of MUC16 in the upper panel.

To determine whether MUC16 affects TRAIL signaling, we knockdown MUC16 cell surface expression. The cell surface localization of MUC16 was confirmed by co-localization with membrane protein Caveolin1 (Additional file 1: Figure S1). We thus derived anti-MUC16 single-chain antibodies (scFvs), containing an ER retention signal, and a control scFv (Ctrl scFv), which does not bind to MUC16 [26,27]. These constructs were stably expressed in MUC16-expressing OVCAR3 cells and two independent MUC16 scFv-expressing cell lines were derived (1:9#7 scFv and 1:9#9 scFv) as previously described [26,27]. As compared to the control scFv, expression of MUC16 scFvs strongly decreased MUC16 expression at the cell surface in OVCAR3 cells as demonstrated by both immunofluorescence and immunoblot (Figure 1). Treatment of control and MUC16 scFv-expressing OVCAR3 cells with increasing concentrations (0 to 100 ng/ml) of TRAIL induced dose-dependent cell death (Figure 2A). However, MUC16 knockdown cells were significantly more sensitive to TRAIL-induced cell death. As shown in Figure 2B, following treatment with TRAIL (10 ng/ml), MUC16 knockdown cells were rounded and floated in the medium indicating cell death whereas the control scFv-expressing cells formed a typical healthy epithelioid monolayer. To further confirm that MUC16 knockdown enhances TRAIL-induced cell death, Ctrl scFv- and 1:9#7 scFv-expressing OVCAR3 cells were incubated with an anti-TRAIL-R2 agonist antibody. As shown in Figure 2C, anti-TRAIL-R2 agonist-induced cell death was enhanced in MUC16 knockdown cells. Apoptosis, as measured by % of hypodiploid cells assessed by flow cytometry, was similar for all cell populations in the absence of TRAIL (Figure 2D) suggesting that MUC16 knockdown by itself does not induce apoptosis. However, MUC16 knockdown significantly sensitized OVCAR3 cells to TRAIL-induced apoptosis (*P* < 0,001) (Figure 2D). In concert with these findings, down-regulation of MUC16 increased TRAIL-induced caspase-8 activation as demonstrated by the near complete disappearance of pro-caspase-8 and the appearance of p18 actives fragments after 3 h when compared to control scFv-expressing cells (Figure 2E). Analysis of TRAIL signaling cascade also indicated that cleaved mature forms of caspase-9 (p35) and caspase-3 (p17) were generated in MUC16 knockdown cells within 1 h. In contrast, control scFv-expressing cells displayed a more limited caspase-9 and caspase-3 activation over time in the presence of TRAIL under the same conditions. Caspase activation in control cells was seen only after 3 or 4 h and cleaved active caspase-8 (p18) and caspase-3 (p17) were not observed even after 4 h. The generation of active caspase-3 fragments in MUC16 knockdown OVCAR3 cells was confirmed by measuring the cleavage of fluorogenic caspase-3 substrates (Additional file 1: Figure S2A). Pre-incubation of cells with caspase-8 inhibitor z-IETD-fmk prior to TRAIL treatment (25 ng/ml) had little effect on TRAIL-induced apoptosis in the control scFv-expressing cells but significantly improved the survival of MUC16 knockdown cells (Additional file 1: Figure S2B).

Caspase-8 mediates the cleavage of full length Bid to generate a truncated form (tBid) which translocates to the mitochondria to promote the insertion of Bax into the outer membrane mitochondrial membrane [29,30]. tBid is therefore a key component that links the death receptor pathway to mitochondria-mediated effector caspase activation in type II cells. We thus assessed the impact of MUC16 knockdown on Bid cleavage in OVCAR3 cells. We observed the near complete disappearance of full length Bid within 3 h in MUC16 knockdown cells treated with TRAIL (Figure 2F). In contrast, the disappearance of Bid occurred only after 8 h in control scFv-expressing cells. The cleavage of Bid in MUC16 knockdown cells was almost completely inhibited by pre-incubation with caspase-8 inhibitor z-IETD-fmk (Additional file 1: Figure S2C). To evaluate whether MUC16 also attenuates TRAIL-induced mitochondrial activation, mitochondria membrane integrity was assessed by the uptake of a lipophilic cationic dye. In the absence of TRAIL, there were little or no visible apoptotic mitochondria in both knockdown and control cells as indicated by the absence of green-labelled cells (Figure 2G). The red fluorescence represents dimeric dye that has accumulated in the intact mitochondria membrane. However, compared to control scFv-expressing cells, MUC16 knockdown cells treated with TRAIL displayed substantially more apoptotic mitochondria as shown by a greater number of green-labeled cells indicating the accumulation of dye in the cytoplasm due to the inability of mitochondria to concentrate the dye within their membranes related to a loss of membrane potential. All together, these results indicate that MUC16 attenuates TRAIL-induced apoptosis, activation of caspase-8 and mitochondrial activation.

To further support the role of MUC16 in TRAIL resistance, we examined the expression of MUC16 in nine ovarian cancer cell lines. As shown in Table 1, with the exception of SKOV3 and OVC116 cell lines, the expression of MUC16 correlated with TRAIL sensitivity. More interestingly, MUC16 expression in TRAIL resistant OVCAR3 cells (OVCAR3 R350) was significantly higher than its isogenic sensitive parental cells (Figure 2G). The generation of the OVCAR3 R350 cell line has been previously described by our group [28].

**Table 1:** MUC16 expression and TRAIL sensitivity

**Ovarian cancer cell lines**	**Relative MUC16 surface expression (MFI)**	**TRAIL IC50 (ng/ml)**
OVCAR3	527	10
OVCAR3 R350	1250	> 500
SKOV3	< 10	> 500
CaOV3	49,6	20
OVC116	< 10	25
OVC118	325	125
COV2	628	> 500
COV17	842	> 500

### MUC16 C-terminal domain (CTD) is sufficient to attenuate TRAIL-induced apoptosis and signaling

**Figure 3:**
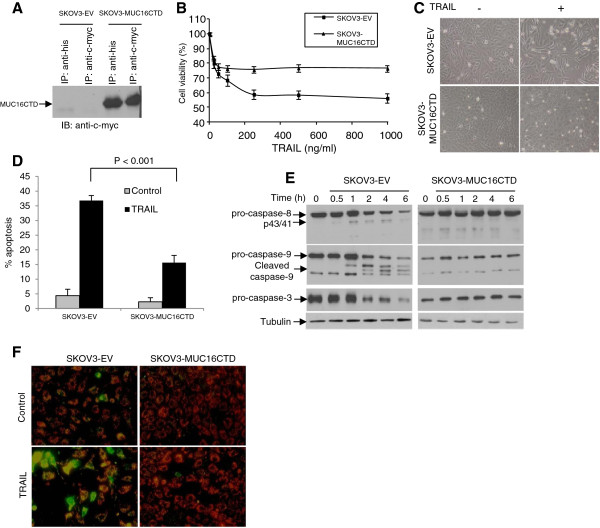
**MUC16CTD is sufficient to attenuate TRAIL-induced apoptosis and caspase-8 activation in SKOV3 cells. (A)** Lysates from SKOV3-EV or SKOV3-MUC16CTD were immunoprecipitated with anti-His or anti-c-myc-789 antibodies and subjected to immunoblotting with anti-c-myc 9E10 antibody. **(B)** SKOV3 cells expressing MUC16CTD or the empty vector (EV) were treated for 48 h with increasing concentrations of TRAIL. Cell viability was measured by the XTT assay and the percentage of cell viability was defined as the relative absorbance of treated versus untreated cells. All assays were performed in triplicate and repeated three times, and data are expressed as the mean of triplicate samples: *bars*, ± SEM. **(C)** Morphological analysis under phase contrast microscopy of SKOV3 cells treated with TRAIL (500 ng/ml) was also performed. Representative fields of one of three independent experiments are shown. **(D)** SKOV3-EV or SKOV3-MUC16CTD cells were incubated in the presence or absence of 500 ng/ml TRAIL and apoptosis was assessed by flow cytometry by analyzing the subG0/G1 DNA content. The percentage of apoptotic cells is indicated. Results from two independent experiments are shown. **(E)** SKOV3-EV and SKOV3-MUC16CTD cells were treated with TRAIL (500 ng/ml) for up to 6 h and cell lysates were analyzed by immunoblots using anti-caspase-8, anti-caspase-3 and anti-caspase-9 antibodies. The pro-caspase forms and their cleavage products are indicated by the arrows to the left of the panels. **(F)** Fluorescence microscopic analysis of the mitochondrial membrane permeability in SKOV3 cells. SKOV3-EV and SKOV3-MUC16CTD cells were cultured for 24 h without TRAIL and the mitochondrial membrane integrity was assessed using the MitoLight™. In treated cells, fresh culture medium containing 500 ng/ml of TRAIL was added for 60 min prior to MitoLight™ staining.

To determine whether the MUC16CTD is sufficient to attenuate TRAIL-induced apoptosis, the MUC16 negative SKOV3 cell line was transfected to stably express an empty vector (SKOV3-EV) or MUC16CTD as previously described [26,27]. MUC16CTD expression was detectable in SKOV3-MUC16CTD cells but not in control SKOV3-EV cells (Figure 3A). The appropriate cell surface localization of MUC16CTD in these cells has been validated previously [27]. Because we have previously shown that SKOV3 cells are less sensitive to TRAIL than OVCAR3 cells [7], higher doses of TRAIL were used for the treatment of SKOV3 cells. As shown in Figure 3B and C, TRAIL-induced cell death was significantly decreased in SKOV3 cells expressing MUC16CTD compared to empty vector (EV)-expressing cells (*P* < 0.001). TRAIL-induced apoptosis was decreased by about 50% in MUC16CTD-expressing SKOV3 cells compared to control SKOV3-EV cells (Figure 3D).

In SKOV3-EV cells, TRAIL induced the cleavage of pro-caspase-8 into the active p43/41 after about 1 h (Figure 3E). Because these cells are less sensitive to TRAIL than OVCAR3 cells, the mature active p18 fragments were not detected in SKOV3-EV cells even after 6 h. The cleavage of caspase-3 and caspase-9 was evident after 2 h as determined by the disappearance of pro-caspase form (Figure 3E). The expression of MUC16CTD markedly decreased the TRAIL-induced processing of caspase-8, caspase-3 and caspase-9 in SKOV3 cells. There was no evidence of pro-caspase cleavage in these cells even after 6 h (Figure 3E). To determine whether TRAIL-induced activation of the mitochondrial pathway was attenuated by MUC16CTD, mitochondria membrane integrity was assessed by the uptake of a lipophilic cationic dye. Experiments showed that MUC16CTD strongly decreased the number of apoptotic mitochondria (Figure 3D). All together, these findings indicate that MUC16CTD is sufficient to inhibit TRAIL-induced caspases activation and apoptosis.

### MUC16 decreases TRAIL-R2 expression and its recruitment at the DISC

**Figure 4:**
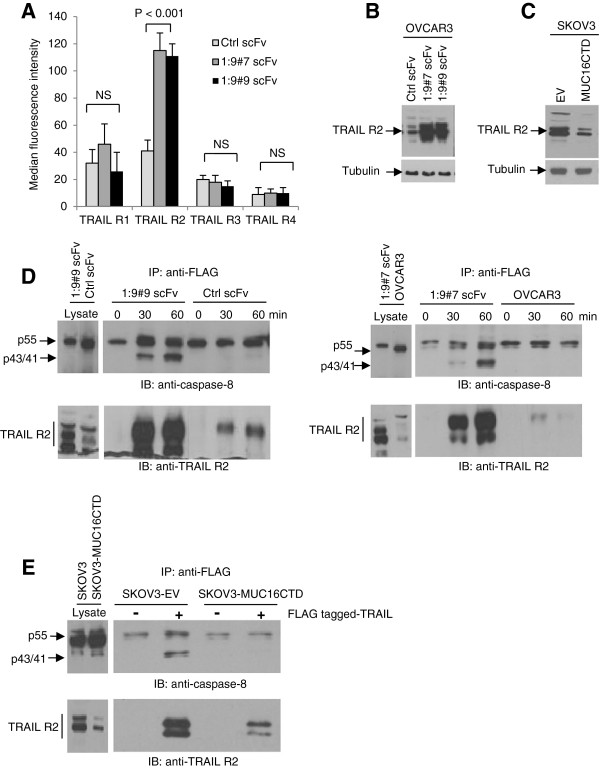
**MUC16 decreases TRAIL-R2 expression and blocks caspase-8 activation at the DISC. (A)** Monolayers of Ctrl scFv- and MUC16 scFv-expressing cells were detached using EDTA, washed with PBS and fixed with paraformadehyde 4% in PBS. Cells (non-permeabilized) were incubated with anti-TRAIL receptors R1, R2, R3 and R4 antibodies. Expression of TRAIL receptors was determined by flow cytometry and expressed as median fluorescence units. Results are from three independent experiments. **(B)** Lysates from Ctrl scFv- and MUC16 scFv-expressing OVCAR3 cells were immunoblotted with TRAIL-R2 antibody. **(C)** Lysates from empty vector (EV)- and MUC16CTD-expressing SKOV3 cells were immunoblotted with TRAIL-R2 antibody. **(D)** Ctrl scFv- and MUC16 scFv-expressing OVCAR3 cells were incubated with FLAG-tagged TRAIL (1 μg/ml) for various times and the DISC was immunoprecipitated with anti-FLAG antibody. The membrane was immunoblotted with anti-caspase-8 antibody or with anti-TRAIL R2. Data are representative of at least two independent experiments. **(E)** Empty vector (EV)- and MUC16CTD-expressing SKOV3 cells were incubated with FLAG-tagged TRAIL (2 μg/ml) and the DISC was immunoprecipitated with anti-FLAG antibody. The membrane was immunoblotted with anti-caspase-8 antibody or with anti-TRAIL R2. Data are representative of at least two independent experiments.

Differential sensitivity to TRAIL-induced apoptosis in tumor cells has been previously attributed to differential expression of death and decoy receptors [30]. Indeed, the levels of TRAIL receptors (TRAIL-R1 and -R2) are closely related to apoptosis induced by TRAIL. It is therefore possible that MUC16 regulates the TRAIL signaling cascade by altering the expression of TRAIL receptors. In this context, we examined the cell surface expression of the four TRAIL receptors by flow cytometry in control and MUC16 knockdown cells. TRAIL-R2 expression was significantly increased in MUC16 knockdown OVCAR3 cells whereas the expression of the other TRAIL receptors remained unchanged (Figure 4A). In concert with these results, the assessment of TRAIL-R2 by immunoblot showed increased expression of TRAIL-R2 in MUC16 knockdown cells (Figure 4B). In contrast, the expression of MUC16CTD was sufficient to decrease TRAIL-R2 expression in SKOV3 cells (Figure 4C).

Binding of TRAIL to TRAIL-R1/TRAIL-R2 results in recruitment of FADD to the DISC and, in turn, FADD recruits pro-caspase-8 [8]. Because MUC16 decreases TRAIL-R2 expression, we determine whether MUC16 affects DISC formation. The composition of TRAIL DISC was analyzed by immunoprecipitation and immunoblotting using cross-linked recombinant Flag-tagged soluble TRAIL. In MUC16 knockdown OVCAR3 cells, pro-caspase-8 activation at the DISC, as demonstrated by the presence of p43/41 fragments, was clearly enhanced when compared to the control scFv-expressing cells (Figure 4D). Immunoblot analysis further revealed increased TRAIL-R2 recruitment at the DISC in MUC16 knockdown cells (Figure 4D) which is consistent with the increased expression of TRAIL-R2 in these cells (Figure 4A and B). In line with experiments in OVCAR3 cells and those shown in Figure 4C, fewer TRAIL-R2 receptors were pulled down by immunoprecipitating flag-tagged TRAIL from SKOV3 MUC16CTD cells and cleaved caspase-8 fragments (p43/41) were detected only in the DISC of control SKOV3-EV cells after 60 min (Figure 4E). These data suggest that MUC16 attenuates TRAIL-induced apoptosis by decreasing TRAIL-R2 expression and consequently decreasing its recruitment at the DISC.

### MUC16 regulates cFLIP expression

**Figure 5:**
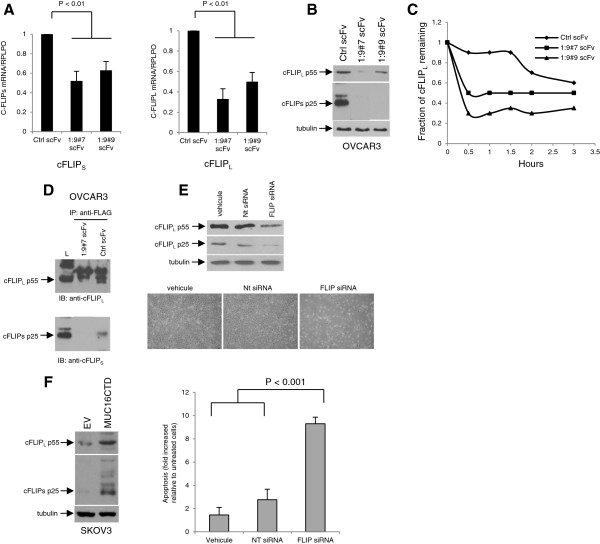
**MUC16 increases cFLIP expression to attenuate TRAIL-induced apoptosis. (A)** Real-time PCR analysis of cFLIP_S_ and cFLIP_L_ transcript levels in Ctrl scFv and MUC16 scFv-expressing OVCAR3 cells. Results were standardized using primers of the housekeeping gene RPLPO. Data are expressed as fold change relative to levels observed in Ctrl scFv-expression OVCAR3 cells. Data are from two independent experiments. **(B)** Lysates from Ctrl scFv- and MUC16 scFv-expressing OVCAR3 cells were immunoblotted with cFLIP_L_ and cFLIP_S_ antibodies. **(C)** OVCAR3 Ctrl scFv and OVCAR3 MUC16 1:9#7 and 1:9#9 scFv were incubated with cycloheximide (100 μM). Lysate were obtained at different time points and immunoblot analysis was performed with anti-cFLIP_L_ antibody. Intensity of cFLIP_L_ signal was determined by densitometric scanning and plotted as the percentage of cFLIP_L_ remaining compared with the control (0 h). **(D)** OVCAR3 Ctrl scFv and OVCAR3 MUC16 1:9#7 scFv were incubated with FLAG-tagged TRAIL (1 μg/ml) and the DISC was immunoprecipitated with anti-FLAG M2 antibody. The membrane was immunoblotted with anti-cFLIP_L_ and with anti-cFLIP_s_ antibodies. **(E)** OVCAR3 cells were transfected with control nontargeting siRNA (NT siRNA) or cFLIP siRNA pools and then incubated with TRAIL. Immunoblot analysis (24 h) was performed with anti-cFLIP_L_ and anti-cFLIP_S_ antibodies and showed decreased cFLIP_L_ and cFLIP_S_ expression in cFLIP siRNA-transfected cells. Apoptosis was qualitatively assessed by phase contrast microscopy in TRAIL treated cells (100 ng/ml; 6 h) and showed morphological changes suggestive of apoptotic cells in cFLIP siRNA-transfected cells. Representative fields from one of two independent experiments are shown. Apoptosis was also by ELISA as described in Methods and expressed as fold increased relative to control (untreated) cells with the mean of triplicates from three independent experiments ± SD. **(F)** Lysates from empty vector- and MUC16 CTD-expressing SKOV3 cells were immunoblotted with cFLIP_L_ and cFLIP_S_ antibodies.

The cellular FLICE inhibitory protein (cFLIP) regulates both recruitment and processing of pro-caspase-8 within the DISC [13,14]. Although several cFLIP isoforms have been described, the two main isoforms expressed in human cells are cFLIP_L_ (55 kDa) and cFLIP_s_ (25 kDa) [13,14]. Because both cFLIP_L_ and cFLIP_S_ are key regulators of the TRAIL signaling cascade, we first assessed whether MUC16 affects the transcription of cFLIP_L_ and cFLIP_S_. We thus examined cFLIP_L_ and cFLIP_S_ mRNA levels in control and MUC16 scFv-expressing OVCAR3 cells. cFLIP_L_ and cFLIP_S_ mRNA levels, as determined by quantitative real time PCR, were significantly decreased in MUC16 knockdown OVCAR3 cells compared to control scFv expressing cells (Figure 5A). In concert with these findings, the expression of both cFLIP_L_ and cFLIP_S_ were decreased in MUC16 knockdown cells when assessed by immunoblot (Figure 5B). To determine if MUC16 affects cFLIP_L_ stability, we blocked *de novo* protein synthesis with cycloheximide and assessed cFLIP_L_ and cFLIP_S_ expression at different times after the addition of cycloheximide. Densitometric scanning of the signals showed that the estimated half-lives of cFLIP_L_ in control scFv- and MUC16 scFv-expressing OVCAR3 cells are > 3 and ≤ 0.5 hours, respectively (Figure 5C). The half-live of cFLIP_S_ was estimated to be ≤ 0.5 hours in control scFv-expressing OVCAR3 cells (data not shown). Because of the very low expression of cFLIP_S_ in MUC16 knockdown cells, its half-live could not be determined using this approach. Nonetheless, these data indicate that MUC16 stabilizes cFLIP_L_ which might contribute to attenuate TRAIL-induced apoptosis in MUC16 expressing malignant cells. Indeed, cFLIP_L_ and cFLIP_S_ recruitment at the DISC were both decreased in MUC16 knockdown cells as compared to control scFv-expressing cells (Figure 5D). In addition, silencing cFLIP in OVCAR3 cells was associated with increased apoptosis in response to TRAIL (Figure 5E). Consistent with these findings, the expression of MUC16CTD in SKOV3 cells was associated with the up-regulation of cFLIP_L_ and cFLIP_S_ as demonstrated by immunoblot (Figure 5F). Of note, the expression of other key regulators of the TRAIL signaling cascade such as Bcl-2, Bcl-XL, Bax, FADD and XIAP were unaffected by MUC16 (Additional file 1: Figures S2D and S2E). These data indicate that MUC16 transcriptionally increases both isoforms of cFLIP and at least cFLIP_L_ protein, thereby increasing their recruitment at the DISC to attenuate TRAIL-induced apoptosis.

## Discussion

MUC16 is a mucin protein overexpressed by most human ovarian epithelial cancers [19,31,32]. Moreover, a correlation exists between rising and falling levels of serum MUC16 and clinical progression and regression of the disease [31]. In this context, it is considered to be one of the most useful clinical markers for ovarian cancer. Although its biological functions remain largely unknown, MUC16 expression in EOC cells has been associated with a more aggressive phenotype [26]. In addition, our group has recently implicated MUC16 in the regulation of genotoxic drug-induced apoptosis but the mechanisms associated with the effect were unclear [27]. In the present study, we demonstrate that MUC16 is involved in the regulation the death receptor pathway. Cell surface MUC16 knockdown, mediated by anti-MUC16 scFvs, sensitizes ovarian cancer cell line OVCAR3 to apoptosis in response to TRAIL, a member of the TNF family of cytokines that represents a promising candidate for cancer treatment because of its ability to selectively induce apoptosis in malignant cells [5]. TRAIL-induced activation of initiator caspase-8 and effector caspase-3 was enhanced in MUC16 knockdown cells. MUC16 knockdown was associated with increased TRAIL-R2 expression and recruitment at the DISC. MUC16 knockdown also decreases the expression of the caspase-8 inhibitor cFLIP at the transcriptional level and enhanced the protein degradation of cFLIP_L_ isoform, thereby enhancing apoptosis in response to TRAIL. Conversely, MUC16CTD expression attenuated TRAIL-induced apoptosis and caspase activation by decreasing TRAIL-R2 expression and recruitment at the DISC and by increasing cFLIP expression. These data suggest that MUC16 plays an important role in regulating the death receptor signaling cascade.

Mucins play a role in carcinogenesis by promoting anchorage-independent growth and tumorigenicity of human epithelial cells. Altered expression of mucins has been associated with increased invasiveness, enhanced tumor cell growth and metastasis, and modulation of cell adhesion in carcinomas [32-38]. Based on its similarity of structure with mucins, it is conceivable that MUC16 exerts a number of functions that parallel those of other mucins. Interestingly, it has been reported that MUC1 can block death receptor-mediated apoptosis in breast and colonic carcinoma cells [39,40]. This effect has been attributed to localization of MUC1 C-terminal domain to the mitochondria which attenuated mitochondrial activation [40] or to the ability of MUC1 to directly bind caspase-8 and FADD, thereby inhibiting recruitment of caspase-8 at the DISC [39]. Thus, similar to MUC1, MUC16 attenuates TRAIL-induced apoptosis in EOC cells and both mucins may act upstream of the mitochondria. However, the mechanisms by which they inhibit TRAIL signaling differ. MUC16 acts through multiple mechanisms to attenuate TRAIL-induced apoptosis. MUC16 down-regulates TRAIL-R2 and up-regulates cFLIP expression both resulting in the inhibition of caspase-8 activation at the DISC. The basis for this discrepancy in term of mechanisms between MUC1 and MUC16 is unclear but may relate to the fact that different cellular models were used or, more likely, to the fact that MUC1 and MUC16 share very little sequence homology in their cytoplasmic tail [41]. MUC16 has a very short intracellular domain (31 a.a.) compared to MUC1 (72 a.a.). Nonetheless, collectively the data suggest that transmembrane mucins, such as MUC1 and MUC16, regulate the extrinsic pathway of apoptosis. By doing so, mucins may enable tumor cells to escape the cytotoxic effect of immune cells and promote tumor development.

Our study provides important insight into the molecular mechanisms by which MUC16 regulates TRAIL-mediated apoptosis. TRAIL binds to distinct receptors but among these receptors, only TRAIL-R1 and TRAIL-R2 contain cytoplasmic death domains that can transduce an apoptotic signal upon TRAIL binding. Our studies show that although both TRAIL-R1 and TRAIL-R2 are expressed at the surface of OVCAR3 and SKOV3 cell populations, TRAIL-R2 appears to be the main receptors used by TRAIL to transduce a death signal in these cells. This is supported by the observation MUC16 knockdown enhances anti-TRAIL-R2 agonist-mediated apoptosis. Although the precise mechanism by which MUC16 alters TRAIL-R2 expression selectively remains to be elucidated, MUC16 expression was associated with a lower expression of TRAIL-R2. Lack or low expression of TRAIL-R2 has been previously associated with TRAIL resistance [30]. Reduced TRAIL-R2 expression on the cell surface may result from either a lower level of expression in the cell or failure to deliver the receptor to the cell surface. Western blotting showed that the total protein levels of TRAIL-R2 are significantly lower in MUC16 expressing cells. This observation does not suggest a defective transport or a trafficking problem as we would expect identical levels of TRAIL-R2 in MUC16-expressing and control cells. A more likely explanation would be a regulation of TRAIL-R2 at the transcriptional levels or increased protein degradation. Microarray analysis to investigate the global changes in MUC16 knockdown OVCAR3 cells revealed a significant (2.71) up-regulation of TRAIL-R2 gene but not TRAIL-R1 (unpublished data). We are currently investigating the mechanisms by which MUC16 regulates TRAIL-R2 expression. Markedly increased pro-caspase-8 cleavage was seen within the DISC of MUC16 knockdown OVCAR3 cells while pro-caspase-8 activation into the DISC was not detected in the SKOV3 ectopically expressing MUC16CTD. Furthermore, OVCAR3 cells endogenously expressing high levels of MUC16 displayed very little pro-caspase-8 processing after incubation with TRAIL for 1 h (Figure 4D). After initial cleavage of pro-caspase-8 at the DISC, active caspase-8 may directly activate effector caspases such as caspase-3 or may alternatively activate the cleavage and translocation of the pro-apoptotic protein Bid to the mitochondria [42]. The activation of the mitochondrial apoptotic pathway leads to cytochrome c release, caspase-9 and caspase-3 activation [42]. Kinetic analysis of caspase-9 and caspase-3 activation in OVCAR3 showed that the mitochondrial pathway is activated regardless of the cell’s MUC16 status. However the activation of this pathway is markedly enhanced in MUC16 knockdown cells (Figure 3E). Interestingly, addition of a caspase-9 inhibitor abrogated this effect indicating that mitochondrial amplification of TRAIL death signaling is important in MUC16 knockdown cells (data not shown). Based on our results, we propose that MUC16 interferes with the caspase-8 activation at the DISC and consequently affects initiation of the mitochondrial amplification loop that leads to TRAIL-induced apoptosis.

It was recently shown that MUC16 silencing in breast cancer cells is associated with up-regulation of TRAIL-R1 (DR4) and pro-apoptotic molecules Bid and Bax, and down-regulation of Bcl-2 [43]. The authors speculated that these changes might promote TRAIL-induced apoptosis in breast cancer cells but the level of the blockade in the death receptor signaling cascade was not established. In EOC cells, neither the expression of MUC16CTD nor MUC16 knockdown was associated with altered expression of Bid, Bax, Bcl-2 or TRAIL-R1, and the blockade occurred upstream of the mitochondria. The basis for the discrepancy between these results is not clear. The role of anti-apoptotic proteins such as Bcl-2 and Bcl-XL in protecting from drug-induced apoptosis has been shown to be cell context dependent [44].

## Conclusions

In summary, our results indicated that MUC16 attenuates TRAIL-induced apoptosis in EOC cells. Further, MUC16 down-regulates TRAIL R2 expression and recruitment at the DISC. MUC16 also increase the expression of both cFLIP_L_ and cFLIP_S_. Altogether, these effects contribute to the MUC16-mediated attenuation of TRAIL-induced apoptosis. The present findings may also have therapeutic implications. In this context, a combination of TRAIL with small molecules that block cell surface localization or the function of the intracellular domain of MUC16 might prove more effective in killing TRAIL resistant ovarian cancer cells. A clearer understanding of the mechanisms underlying MUC16 intracellular signaling could lead to the development of novel approaches to enhance the efficacy of TRAIL for the treatment of ovarian cancer.

## Competing interests

The authors report no declarations of interest.

## Authors’ contributions

IM participated in the design of the study and performed most of the experiments. DL performed the TRAIL sensitivity assays and some of the assays for measuring apoptosis. MB generated and validated the MUC6CTD-expressing SKOV3 cells. CR participated in the design of the study and helped to draft the manuscript. AP conceived the study, participated in its design and drafted the manuscript. All authors read and approved the final manuscript.

## Pre-publication history

The pre-publication history for this paper can be accessed here:

http://www.biomedcentral.com/1471-2407/14/234/prepub

## Supplementary Material

Additional file 1: Figure S1 Expression and localization of MUC16 in OVCAR3 cells. Immunofluorescence studies were performed as described in Methods. MUC16 and caveolin1 were visualized with Texas Red (MUC16) or Alexa fluorescent label (green; caveolin1). Representative images are shown. Scale = 100 μM. Data demonstrate that MUC16 co-localization at the cell membrane with caveolin1. **Figure S2.** MUC16 enhanced TRAIL-induced Bid cleavage and mitochondrial activation. (a) The parental OVCAR3 cell line and sublines (ctrl scFv, 1:9#7 scFv and 1:9#9 scFv) were treated with TRAIL (100 ng/ml) and caspase-3 activity was measured using a caspase-3 fluorogenic protease assay. In brief, after TRAIL treatment, cells were lysed in 250 μl of lysis buffer and lysates were incubated with 50 μM of DEVD-AFC substrate for 1 h. Caspase-3 activity was measured using the Versa Fluor fluorometer (b) Parental NIH:OVCAR3 cells and sublines (ctrl scFv, 1.9#7 and 1.9#9) were treated with TRAIL (100 ng/ml) for 6 h. The activity of caspase-3 was measured using a caspase-3 fluorogenic protease assay with 50 μM of DEVD-AFC as a substrate. Results are expressed as relative fluorescence unit (RFU) of caspase-3 activity normalized for the total amount of protein in the extract and represent mean ± SEM (n = 3). *, indicates *P* < 0.001. Click here for file
