# Supplementary material for: MUC16 mucin (CA125) attenuates TRAIL-induced apoptosis by decreasing TRAIL receptor R2 expression and increasing c-FLIP expression
Source: BMC Cancer. 2014 Apr 1;14:234. doi: 10.1186/1471-2407-14-234 (PMC4234371; doi:10.1186/1471-2407-14-234)
Supplement: Additional file 1: Figure S1 — Expression and localization of MUC16 in OVCAR3 cells. Immunofluorescence studies were performed as described in Methods. MUC16 and caveolin1 were visualized with Texas Red (MUC16) or Alexa fluorescent label (green; caveolin1). Representative images are shown. Scale = 100 μM. Data demonstrate that MUC16 co-localization at the cell membrane with caveolin1. Figure S2. MUC16 enhanced TRAIL-induced Bid cleavage and mitochondrial activation. (a) The parental OVCAR3 cell line and sublines (ctrl scFv, 1:9#7 scFv and 1:9#9 scFv) were treated with TRAIL (100 ng/ml) and caspase-3 activity was measured using a caspase-3 fluorogenic protease assay. In brief, after TRAIL treatment, cells were lysed in 250 μl of lysis buffer and lysates were incubated with 50 μM of DEVD-AFC substrate for 1 h. Caspase-3 activity was measured using the Versa Fluor fluorometer (b) Parental NIH:OVCAR3 cells and sublines (ctrl scFv, 1.9#7 and 1.9#9) were treated with TRAIL (100 ng/ml) for 6 h. The activity of caspase-3 was measured using a caspase-3 fluorogenic protease assay with 50 μM of DEVD-AFC as a substrate. Results are expressed as relative fluorescence unit (RFU) of caspase-3 activity normalized for the total amount of protein in the extract and represent mean ± SEM (n = 3). *, indicates P < 0.001. [file 1471-2407-14-234-S1.ppt]

## Slide 1
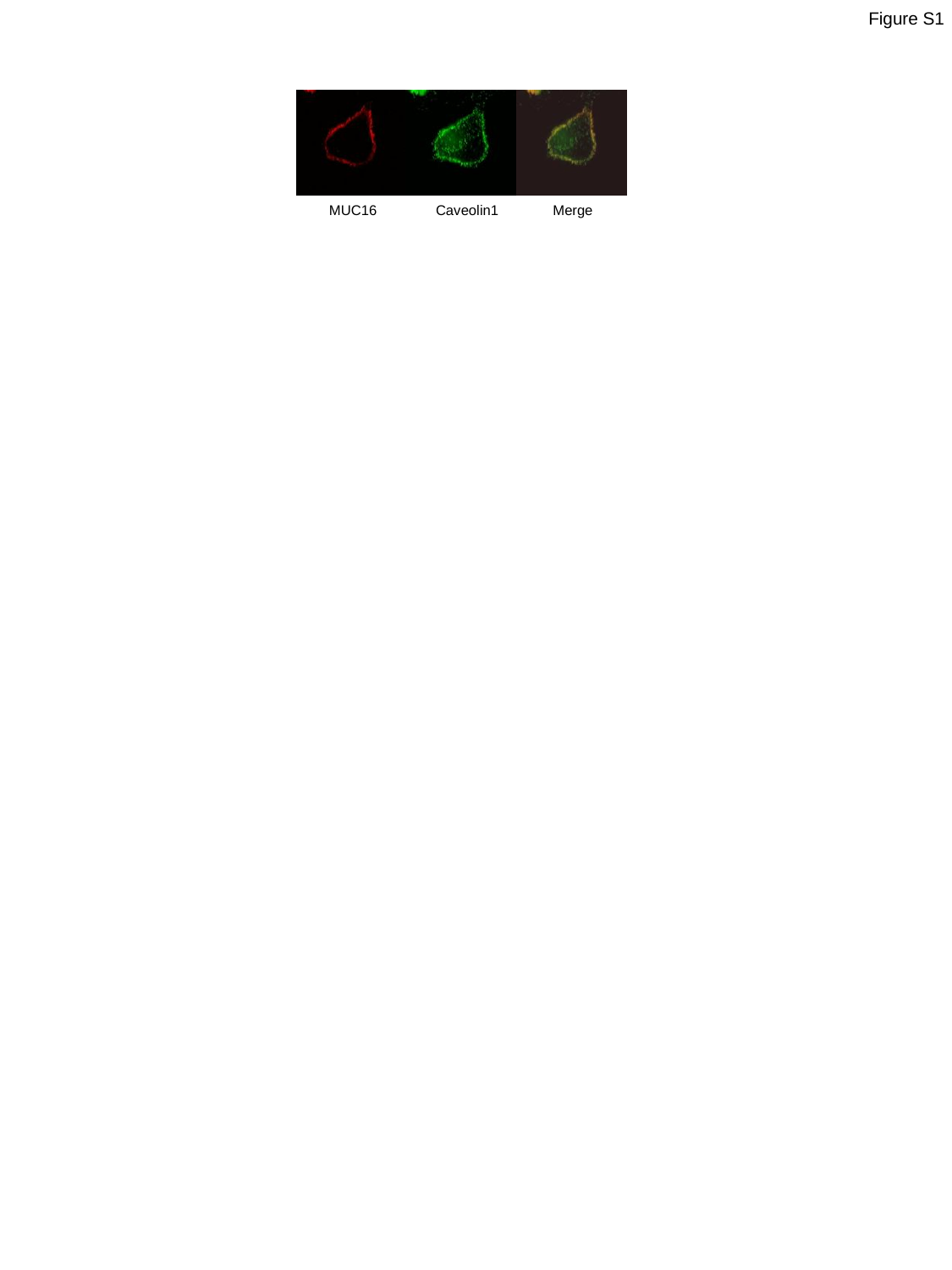

Figure S1
MUC16
Caveolin1
Merge

## Slide 2
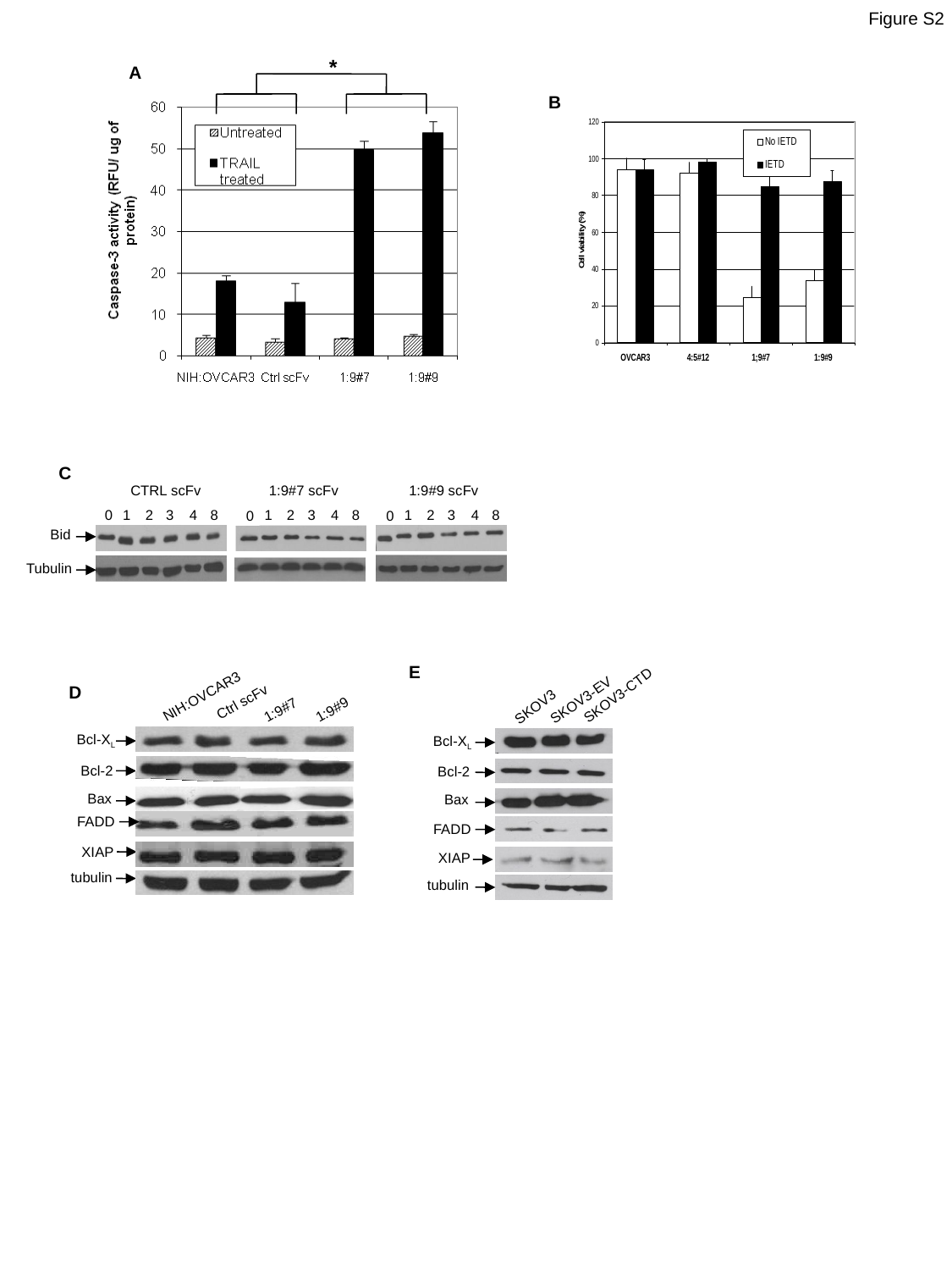

Figure S2
*
A
B
C
CTRL scFv
1:9#7 scFv
1:9#9 scFv
1
2
3
4
0
8
1
2
3
4
8
1
2
3
4
8
0
0
Bid
Tubulin
E
SKOV3-CTD
D
NIH:OVCAR3
SKOV3-EV
Ctrl scFv
SKOV3
1:9#9
1:9#7
Bcl-XL
Bcl-XL
Bcl-2
Bcl-2
Bax
Bax
FADD
FADD
XIAP
XIAP
tubulin
tubulin
